# Cofilin Inhibitor Protects against Traumatic Brain Injury-Induced Oxidative Stress and Neuroinflammation

**DOI:** 10.3390/biology12040630

**Published:** 2023-04-21

**Authors:** Ghaith A. Bahader, Antonisamy William James, Daniyah A. Almarghalani, Zahoor A. Shah

**Affiliations:** 1 Department of Medicinal and Biological Chemistry, The University of Toledo, 3000 Arlington Avenue, Toledo, OH 43614, USA; 2 Department of Pharmacology and Experimental Therapeutics, College of Pharmacy and Pharmaceutical Sciences, The University of Toledo, Toledo, OH 43614, USA

**Keywords:** cofilin inhibitor, TBI, oxidative stress, microglial activation, ROS, Nrf2

## Abstract

**Simple Summary:**

Traumatic brain injury (TBI) is a significant healthcare problem and a leading cause of death in the United States. There is a critical need to develop potential therapeutics to treat TBI-related injuries. Oxidative stress is considered a major mechanism that worsens the damage. Microglia, the first line of defense in the brain, is overactivated following injury causing the death of neuronal cells. Cofilin is a cytoskeleton protein that is activated during such brain injuries. As reported in previous in vivo studies, targeting cofilin has already been shown as a promising therapeutic strategy in other brain diseases. This study investigated the potential benefits of a new cofilin inhibitor in reducing microglial cell activation and the death of neurons in in vitro immortalized cells. We also explored this cofilin inhibitor’s effect in a mouse TBI model. Following brain injury, we measured the levels of different genes and proteins in mice brains. We found that the administration of cofilin inhibitor reduced various inflammatory and oxidative markers in in vitro and in vivo mice models.

**Abstract:**

Microglial activation and failure of the antioxidant defense mechanisms are major hallmarks in different brain injuries, particularly traumatic brain injury (TBI). Cofilin is a cytoskeleton-associated protein involved in actin binding and severing. In our previous studies, we identified the putative role of cofilin in mediating microglial activation and apoptosis in ischemic and hemorrhagic conditions. Others have highlighted the involvement of cofilin in ROS production and the resultant neuronal death; however, more studies are needed to delineate the role of cofilin in oxidative stress conditions. The present study aims to investigate the cellular and molecular effects of cofilin in TBI using both in vitro and in vivo models as well as the first-in-class small-molecule cofilin inhibitor (CI). An in vitro H_2_O_2_-induced oxidative stress model was used in two different types of cells, human neuroblastoma (SH-SY5Y) and microglia (HMC3), along with an in vivo controlled cortical impact model of TBI. Our results show that treatment with H_2_O_2_ increases the expression of cofilin and slingshot-1 (SSH-1), an upstream regulator of cofilin, in microglial cells, which was significantly reduced in the CI-treated group. Cofilin inhibition significantly attenuated H_2_O_2_-induced microglial activation by reducing the release of proinflammatory mediators. Furthermore, we demonstrate that CI protects against H_2_O_2_-induced ROS accumulation and neuronal cytotoxicity, activates the AKT signaling pathway by increasing its phosphorylation, and modulates mitochondrial-related apoptogenic factors. The expression of NF-E2-related factor 2 (Nrf2) and its associated antioxidant enzymes were also increased in CI-treated SY-SY5Y. In the mice model of TBI, CI significantly activated the Nrf2 and reduced the expression of oxidative/nitrosative stress markers at the protein and gene levels. Together, our data suggest that cofilin inhibition provides a neuroprotective effect in in vitro and in vivo TBI mice models by inhibiting oxidative stress and inflammatory responses, the pivotal mechanisms involved in TBI-induced brain damage.

## 1. Introduction

Traumatic brain injury (TBI) is a significant cause of death and long-term disability in the United States. An estimated 1.5 million individuals are suffering from TBI each year, with (230,000 hospitalized and 50,000 death) [1], with the major cause being sports, vehicle accidents, and war [2,3]. Moreover, TBI has an economic burden on society, with an estimated annual cost of USD 48.6 billion in the United States alone [4]. Although no effective therapeutics can alleviate TBI-associated brain damage and neurological impairment, preclinical studies have shown that targeting key pathological mechanisms is an effective approach [5]. Microglial activation, neuroinflammation, and oxidative stress have been involved in the pathophysiology of several neurodegenerative diseases including TBI [6,7]. Oxidative stress is caused by the imbalance between the antioxidant defense and the production of oxygen-derived radicals such as hydrogen peroxide (H_2_O_2_), superoxide, and nitric oxide, leading to the excessive generation of reactive oxygen species (ROS) [8]. Excessive ROS production and accumulation represent a major hallmark in the pathogenesis of TBI that can damage the structure of cell membranes and other key cellular molecules such as lipids, proteins, and DNA, which eventually disrupt cellular integrity [9]. Among the various species, H_2_O_2_-induced ROS production and oxidative stress response can lead to mitochondrial dysfunction and initiate cellular apoptosis in different types of cells [10]. H_2_O_2_-induced mitochondrial dysfunction and cell apoptosis are partially regulated by the expression of pro-apoptotic BAX and anti-apoptotic Bcl-2 proteins [11]. Studies have shown that activating antioxidant cellular defense systems may prevent oxidative stress-induced neuronal apoptosis in different neurological diseases [12,13]. The nuclear factor erythroid 2–related factor 2 (Nrf2) is a major regulator of cellular resistance to oxidative stress. It regulates the cellular response to oxidative damage by mediating the expression of antioxidant enzyme genes such as heme oxygenase 1 (HO-1), sodium oxide dismutase 2 (SOD2), NAD(P)H quinone oxidoreductase 1 (NQO1), and glutathione peroxidase (GPx) [14]. 

Often referred to as “CNS resident macrophage”, microglia play an important role in the pathogenesis of several neurodegenerative diseases. Although it has some beneficial functions, such as clearance of cellular debris following brain injury, excessive microglia activation is deleterious and aggravates secondary brain injury [15]. Activated microglia mediates brain neuroinflammation and subsequent neurotoxicity by releasing proinflammatory cytokines and cytotoxic substances [16]. A major transcription factor controlling proinflammatory gene expression in microglial cells, nuclear factor-κB (NF-κB), is activated by exogenous H_2_O_2_ application or endogenous H_2_O_2_ release, such as through the activation of NADPH oxidase (NOX) enzymes. The activation of NF-κB induces an inflammatory phenotype of microglia, which is exhibited by increased expression of inflammatory mediators such as tumor necrosis factor α (TNFα), interleukin-1β (IL-1β), and high mobility group box1 (HMGB1) [17,18].

Cofilin is an actin-associated protein that regulates the dynamics of the actin cytoskeleton through binding, depolarization, and severing of filamentous actin (F-actin) into globular actin (G-actin) [19]. The activity and dynamics of cofilin are regulated by its phosphorylation/dephosphorylation process by various kinases and phosphatases. LIM kinase mediates cofilin phosphorylation at ser3 and the formation of phospho-cofilin (p-cofilin), while slingshot phosphatase 1 (SSH1) mediates its dephosphorylation [20]. Evidence suggests that cofilin activation and the formation of cofilin-actin rods trigger the initiation of apoptosis [21] and are involved in the pathogenesis of neurodegenerative diseases such as Alzheimer’s and ischemic stroke [22,23]. Using gene-editing techniques such as siRNA, studies from our lab have demonstrated that inhibiting cofilin expression is effective in restoring neuronal viability and attenuating microglial activation in different stressful conditions [16,24,25]. However, using a small molecule that can deliver the same therapeutic outcomes is an integral approach to overcoming challenges associated with gene therapy. A recent report from our lab showed that treatment with a newly synthesized novel small molecule cofilin inhibitor (CI) reduced thrombin-induced microglial activation and neuronal apoptosis in vitro [26]. 

In the present study, we investigated the role of cofilin in TBI by using oxidative stress models of H_2_O_2_-induced microglial activation and neurotoxicity as well as controlled cortical impact representing both in vitro and in vivo TBI models. In addition, we explored the potential therapeutic effects of CI in reducing microglial-related inflammatory markers, neuronal apoptosis, and oxidative stress both in vitro and in vivo. 

## 2. Material and Methods

### 2.1. Cell Culture

The human microglia clone 3 cell line (HMC3) (ATCC, Manassas, VA, USA) was cultured in DMEMF12 (Thermo Scientific, West Palm Beach, FL, USA) supplemented with 5% heat-inactivated fetal bovine serum (FBS), 5% horse serum, 2 mM L-glutamine, 1 mM sodium pyruvate, and 1% penicillin/streptomycin at 37 °C in a humidified atmosphere of 95% air and 5% CO_2_. HMC3 were seeded at a density of 1 × 10^5^ cells in 6-well plates with H_2_O_2_ and H_2_O_2_ + cofilin inhibitor (CI) cultured 24 h for protein expression studies.

SH-SY5Y (neuroblastoma cell line) cells (ATCC, Manassas, VA, USA) were cultured in DMEMF12 (Thermo Scientific) supplemented with 10% heat-inactivated fetal bovine serum (FBS), 2 mM L-glutamine, 1 mM sodium pyruvate and 1% penicillin/streptomycin at 37 °C in a humidified atmosphere of 95% air/5% CO_2_. SH-SY5Y were seeded at a density of 1 × 10^5^ cells in 6-well plates for protein expression studies and a density of 1 × 10^4^ cells in 96-well plates for the viability studies.

### 2.2. Animals and TBI Model

In this study, we used male C57BL/6J (10–12 weeks old) mice obtained from The Jackson Laboratories (Bar Harbor, ME, USA). Mice were housed in the Department of Laboratory Animal Resources (DLAR), University of Toledo, Health Science Campus (HSC) under a 12 h light–dark cycle, with free food and water access. All animal procedures were carried out in accordance with protocols approved by the Institutional Animal Care and Utilization Committee (IACUC) of the University of Toledo and under the guidelines of the National Institute of Health (NIH).

A total of 30 mice were randomly subdivided into three groups (sham, TBI + vehicle, and TBI + CI) and subjected to moderate controlled cortical impact (CCI) or sham surgery. Briefly, mice were anesthetized with isoflurane (3% for induction and 1–2% for maintenance) and placed into the stereotaxic frame. A longitudinal midline incision was made, the skin retracted, and a 4 mm craniectomy was performed over the left hemisphere midway to the lambda and bregma sutures and laterally midway between the central suture and the temporalis muscle. The skull cap was removed carefully without damaging the underlying dura. CCI injury was induced in mice using an impactor with a 3 mm in diameter piston that was angled and placed perpendicular to the exposed cortex. The CCI injury was performed with a velocity of 3 m/s, a depth of 1.5 mm, and a dwelling time of 300 milliseconds. After the injury, the incision was sutured, and the mice were allowed to recover on a heating pad for 30 min. Sham mice underwent the same procedure but without the CCI impact.

### 2.3. MTT Assay

SH-SY5Y cells (1 × 10^4^) were seeded in 96-well plates and after 36 h, cells were treated with different concentrations of H_2_O_2_ (10 to 250 μM) for 24 h; then, 10 μL of CCK-8 reagent was added into each well and incubated at 37 °C for 2 h according to manufactures instruction. The [2-(2-methoxy-4-nitrophenyl)-3-(4-nitrophenyl)-5-(2, 4-disulfophenyl)-2H tetrazolium, monosodium salt] is reduced by dehydrogenase in cells to give an orange-colored product (formazan), which is soluble in the tissue culture medium. The amount of the formazan dye generated by dehydrogenases in cells is directly proportional to the number of living cells. The absorbance was measured at 450 nm using a microplate reader, normalized with control, and calculated as a percentage change of cell viability or death. For the CI experiments, SH-SY5Y cells (1 × 10^4^) were seeded in 96-well plates, and after 36 h, cells were co-treated with vehicle or different concentrations of CI (3, 5, 7, 10, 20 μM) with H_2_O_2_ (200 μM) for 24 h followed by same steps mentioned above.

### 2.4. Western Blotting (WB)

Cells or brain tissue samples were harvested by homogenizing them in an ice-cold RIPA buffer (Thermofisher Scientific, Waltham, MA, USA) supplemented with a protease and phosphatase inhibitor cocktail (Sigma-Aldrich, St. Louis, MO, USA) for 30 min. Supernatants containing the total protein fraction were collected after centrifugation at 14,000× *g* for 15 min. Protein concentration was determined using Bradford reagent (Bio-Rad Laboratories, Hercules, CA, USA) according to the manufacturer protocol. Equivalent amounts of proteins from each sample were loaded onto 12% sodium dodecyl sulfate (SDS)-polyacrylamide gels, separated by electrophoresis, and then transferred to polyvinylidene fluoride (PVDF) membranes. Membranes were blocked with 3% BSA for 1 h to avoid non-specific binding. Next, membranes were incubated overnight at 4 °C with different primary antibodies, including cofilin, p-cofilin, TNFα, NF-κB, SOD2, NQO1, p-Akt, BAX, Bcl-2, caspase-3, cleaved caspase-3, GAPDH, β-actin (Cell Signaling Technology, Danvers, MA, USA), SSH-1, HMGB1 (Abcam, Cambridge, MA, USA), Nrf2, and HO-1 (Santa Cruz Biotechnology, Santa Cruz, CA, USA). The membranes were then washed and incubated with horseradish peroxidase-conjugated goat anti-mouse and anti-rabbit secondary antibodies (Cell Signaling Technology) for 1 h at room temperature. GAPDH and β-actin were used as loading controls. Finally, images were captured using Syngene Imaging System (Frederick, MD, USA), and bands were analyzed using ImageJ software 1.53t (National Institutes of Health, Bethesda, Maryland, USA).

### 2.5. Real-Time Quantitative Polymerase Chain Reaction (RT-qPCR)

Total mRNA was isolated from mice brain tissue samples from the perilesional cortex region using TRIzol reagent (Invitrogen, Carlsbad, CA, USA). Complementary DNA (cDNA) synthesis was conducted using an iScript cDNA synthesis kit (Bio-rad). The mRNA expression level was detected using Advanced Universal SYBR Green Supermix (Bio-Rad) in the Step OnePlus Real-Time PCR System (Thermofisher Scientific). GAPDH was used as a housekeeping control. Relative gene abundance levels were analyzed by double delta Ct analysis 2^−ΔΔCT^. The primer pairs that were used for RT-qPCR measurements are mentioned in Table 1.

### 2.6. DCF-DA ROS Staining

SH-SY5Y cells were seeded in 6-well plates, and after 36 h, cells were co-treated with 200 μM concentration of H_2_O_2_ with or without 10 μM concentration of CI for 24 h. Cells were washed with 1× buffer and then stained with 20 μM DCF-DA for 30 min at 37 °C, and cells were rewashed with 1× buffer to remove overstaining. Live cell imaging was performed using a fluorescence microscope at 20× magnification with fluorescein (FITC) and maintained at low light to avoid photobleaching and photo-oxidation.

### 2.7. Immunohistochemistry

Three days following TBI, mice were anesthetized with Ketamine/Xylazine (100/10 mg/kg, intraperitoneally) and then transcardially perfused with 1× phosphate buffer saline (PBS) followed by 4% paraformaldehyde (PFA). Mice brains were dissected, placed in 4% PFA for 24 h, and paraffin-embedded, and 8 µm sections were made using a microtome and used for the staining. The staining procedure started with deparaffinized brain sections in different gradients of xylene/ethanol, followed by antigen retrieval in a pressure cooker for 15 min, washing with 1× PBS, and blocking in 5% BSA for 2 h at room temperature. The following primary antibodies were added subsequently and incubated overnight at 4 °C: mouse anti-iNOS (1:200, Sigma), and rabbit anti-3NT (1:200, Cell Signaling). After washing with 1× PBS, slides were incubated with secondary antibodies for 1 h at room temperature (Taxes red labeled donkey anti-rabbit IgG and Alexa Fluor donkey anti-mouse IgG) (1:1000; Jackson, Immunoresearch, West Grove, PA, USA). Then, the slides were washed with 1× PBS, mounted with ProLong^®^ Gold Antifade Mountant with DAPI (Molecular Probes), and images were captured using fluorescence microscopy. Fluorescence intensity was analyzed using ImageJ software (NIH, USA).

### 2.8. Drug Treatments/Administration

For in vitro studies, cofilin inhibitor (CI) was dissolved in DMSO to prepare 10 mM stock; then, 3, 5, 7, 10, and 15 μM/mL concentrations were used in corresponding experiments. After stimulating cells with 200 μM H_2_O_2_ and co-treating with 10 μM CI for 24 h, cells were harvested for protein expression studies. For in vivo studies, CI was dissolved in 4.9% DMSO, 4.9% Tween-20, 88.9% solubilizing agent, and the final concentration was 1.3%. The first intravenous injection of CI (25 mg/kg) was given 4 h after the TBI followed by 25 mg/kg intraperitoneal injections every 12 h for three days.

### 2.9. Statistical Analysis

Experimental data were presented as the mean ± SEM and were analyzed using GraphPad Prism software (GraphPad Software, San Diego, CA, USA). Each experiment was repeated three times, and data were analyzed using one-way analysis of variance (ANOVA) followed by the Newman–Keuls multiple comparisons test. Additionally, Student’s unpaired *t* test was used to determine the significance between the two groups. A *p* < 0.05 value was considered significant.

## 3. Results

### 3.1. Cofilin and SSH1 Are Activated in Microglial Cells after H_2_O_2_ Treatment

**Figure 1:**
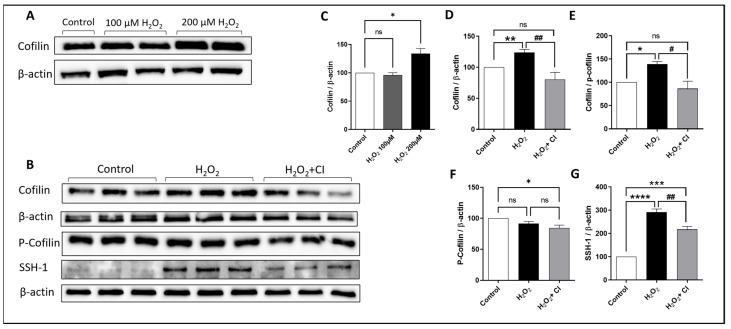
The expression levels of cofilin, p-cofilin, and SSH-1 in response to H_2_O_2_ treatment in microglial cells. HMC3 microglial cells were treated with 100 and 200 μM H_2_O_2_ for 24 h, and then, the protein expression level was evaluated by WB. (**A**,**C**) While there was no change in cofilin expression in the 100 μM H_2_O_2_ treated group, it was significantly upregulated in 200 μM concentration of H_2_O_2_; hence, 200 μM was used for the following experiments. (**B**) Immunoblots of cofilin, p-cofilin and SSH-1 were analyzed by WB. The expression levels of cofilin (**D**), cofilin/p-cofilin ratio (**E**), and SSH1 (**G**) but not p-cofilin (**F**) were significantly increased in the H_2_O_2_ group and significantly reduced when treated with CI. Data are expressed as mean ± SEM of three independent experiments where *p* < 0.05 was considered significant. * *p* < 0.05, ** *p* < 0.01, *** *p* < 0.001, **** *p* < 0.0001 relative to control group. # *p* < 0.05, ## *p* < 0.01 relative to H_2_O_2_ group. (See Figure S1 for original Western blot images).

To investigate the activation of cofilin in microglia after H_2_O_2_ treatment, HMC3 human microglial cells were treated with two doses of H_2_O_2_ (100 μM and 200 μM) for 24 h, and the expression of cofilin was analyzed by WB. Although there was no difference with 100 μM H_2_O_2_ treatment, cofilin expression was significantly increased when treated with 200 μM H_2_O_2_ (Figure 1A,C). Accordingly, 200 μM H_2_O_2_ treatment was used in the subsequent experiments. Next, we tested the effect of CI on inhibiting cofilin activation in H_2_O_2_-treated microglial cells. HMC3 cells were challenged with 200 μM H_2_O_2_ in the presence of 10 μM CI or vehicle for 24 h. WB analysis confirmed the increase in cofilin expression in the H_2_O_2_-treated group; however, treatment with CI significantly reduced the cofilin expression compared to the H_2_O_2_ group (Figure 1D). H_2_O_2_ treatment did not change the expression of p-cofilin (Figure 1F) in the H_2_O_2_ group; however, the cofilin/p-cofilin ratio was significantly reduced in the CI-treated group as compared to the H_2_O_2_ (Figure 1E). Cofilin activity is modulated through phosphorylation/dephosphorylation processes. The activation of cofilin is mediated through dephosphorylation by multiple phosphatases, and SSH1 phosphatase is one of them [27]. Our results showed that SSH1 protein expression was significantly increased in microglial cells treated with H_2_O_2_ as compared to the control, and it was significantly reduced in the CI-treated cultures (Figure 1G). These results indicate that cofilin is activated during oxidative stress conditions induced by H_2_O_2_, which can be inhibited by CI treatment. 

### 3.2. CI Treatment Reduced the H_2_O_2_-Induced Microglial Activation

**Figure 2:**
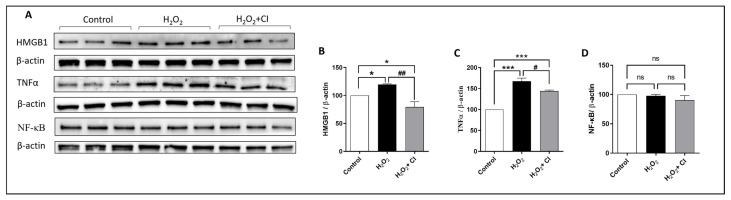
The effect of CI treatment on the H_2_O_2_-induced microglial cell activation. HMC3 microglial cells were treated with 200 μM H_2_O_2_ with or without 10 μM CI for 24 h, and then, the protein expression level was evaluated by WB. (**A**) WB of different proteins in the microglial cell lysate 24 h following H_2_O_2_ treatment. Analysis of WB shows that relative to H_2_O_2_ stimulated control, treatment with CI reduced the H_2_O_2_-induced HMGB1 (**B**) and TNFα (**C**) expression significantly, while there was no significant change in the expression of NF-κB (**D**). Data are expressed as mean ± SEM of three independent experiments, where *p* < 0.05 was considered significant. * *p* < 0.05, *** *p* < 0.001 relative to control group. # *p* < 0.05, ## *p* < 0.01 relative to H_2_O_2_ group. (See Figure S2 for original Western blot images).

ROS, in particular H_2_O_2_, induces microglial proliferation and activation through different regulators, and the NF-κB signaling pathway is one of them. NF-κB is considered a master transcription factor that controls the proinflammatory gene expression in microglia [28]. Proinflammatory substances such as HMGB1 bind to cellular receptors (such as Toll-like receptor 4), which activates NF-κB and the production of inflammatory cytokines such as TNFα. Therefore, our next objective was to understand the impact of CI treatment on H_2_O_2_-induced microglial activation. WB analysis showed that the expression of HMGB1 and TNFα was significantly reduced in the CI-treated cultures compared to the vehicle-treated cultures (Figure 2B,C). Of note, there was no change in the expression level of total NF-κB (Figure 2D).

### 3.3. H_2_O_2_ Activates Cofilin Signaling, and CI Treatment Protects against H_2_O_2_-Induced Neurotoxicity in SH-SH5Y Cells

**Figure 3:**
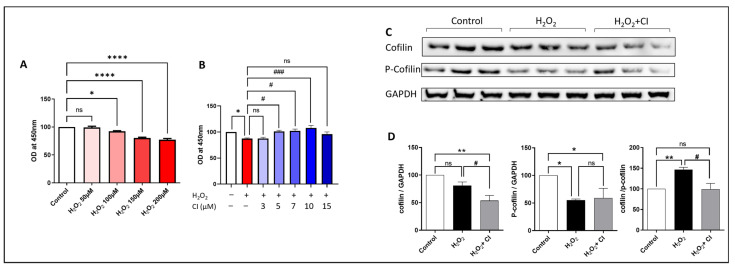
The effect of H_2_O_2_ stimulation and CI treatment on SH-SY5Y cell viability and cofilin signaling. (**A**) SH-SY5Y cells were treated with increasing concentrations of H_2_O_2_ for 24 h followed by the CCK8 viability assay to measure the neurotoxic potential of H_2_O_2_. SH-SY5Y cell viability was significantly reduced with concentrations of 100, 150, and 200 μM of H_2_O_2_. (**B**) SH-SY5Y cells were treated with 200 μm H_2_O_2_ alone or with an increasing concentration of CI for 24 h, and cell viability was measured using the CCK8 assay. Cell viability was significantly reduced in the H_2_O_2_-treated group; however, treatment with 5, 7, and 10 μM CI significantly protected the cells from the H_2_O_2_-induced neurotoxic effect. (**C**,**D**) The expression level of cofilin and p-cofilin was tested by WB. H_2_O_2_ significantly reduced the p-cofilin expression and increased cofilin dephosphorylation, which was rescued by CI treatment. Data are expressed as mean ± SEM of three independent experiments where *p* < 0.05 was considered significant. * *p* < 0.05, ** *p* < 0.01, **** *p* < 0.0001 relative to control group. # *p* < 0.05, ### *p* < 0.001 relative to H_2_O_2_ group. (See Figure S3 for original Western blot images).

Next, we tested the neuroprotective potential of CI against H_2_O_2_-induced cell death using the SH-SY5Y neuroblastoma cell line. CCK8 assay was used to assess the viability of SH-SY5Y cells. First, we treated the cells with increasing concentrations of H_2_O_2_ for 24 h and then measured the cell viability. We found that SH-SY5Y cell viability was remarkably reduced starting from 100 µM H_2_O_2_ concentration, and the reduction is proportionally increased with increased H_2_O_2_ concentration compared to controls (Figure 3A). Accordingly, we decided to choose the 200 µM concentration, which was associated with around 20–25% reduction in cell viability. In the following experiment, we incubated the SH-SY5Y cells with 200 µM H_2_O_2_ plus vehicle or increasing concentration of CI and assessed the cell viability. Our results showed that the viability of SH-SY5Y cells was significantly increased starting from 5 µM concentration of CI with the highest increase with 10 µM concentration; however, there was no increase in the viability of the cells with 3 and 15 µM concentrations of CI as compared to the H_2_O_2_ vehicle-treated cultures (Figure 3B). Accordingly, a concentration of 10 µM CI was used for the followed experiments. Next, we investigated cofilin signaling in SH-SY5Y cells 24 h following stimulation with 200 µM H_2_O_2_ with or without CI. Although the cofilin expression was not significantly changed in the H_2_O_2_-stimulated cells, there was a significant reduction in the expression of p-cofilin (Figure 3C,D). Analyzing the ratio of cofilin to p-cofilin showed a significant increase in the cofilin dephosphorylation state. Moreover, CI treatment significantly reduced the total cofilin and the cofilin/p-cofilin ratio compared to vehicle-treated cells.

### 3.4. CI Treatment Reduces the H_2_O_2_-Induced ROS Production in SH-SY5Y Cells

**Figure 4:**
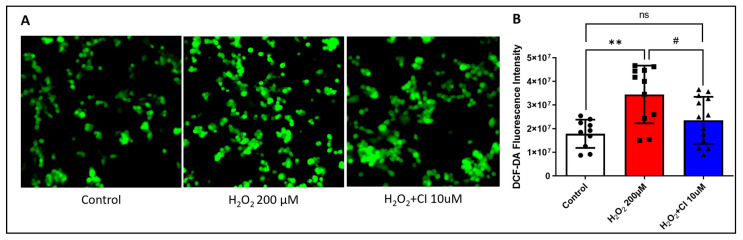
The effect of CI treatment on H_2_O_2_-induced ROS production in SH-SY5Y cells. SH-SY5Y cells were treated with 200 μm H_2_O_2_ with or without 10 μM concentration of CI for 24 h, and ROS level was determined by fluorescence microscopy after DCF-DA staining. (**A**) Representative fluorescence images of DCF-DA staining SH-SY5Y cells. (**B**) Quantifying green fluorescence intensity showed a significant reduction of ROS levels in the CI-treated group compared to the H_2_O_2_-stimulated group. Data are expressed as mean ± SEM of three independent experiments where *p* < 0.05 was considered significant. ** *p* < 0.01 relative to the control group. # *p* < 0.05 relative to H_2_O_2_ group.

ROS generation is an important indicator of oxidative stress and mitochondrial dysfunction in cells, resulting in cell apoptosis. Therefore, to determine the effect of CI in reducing ROS production, SH-SY5Y cells were treated with H_2_O_2_ in the presence of 10 µM CI or vehicle, and ROS level was measured using DCF-DA fluorescence staining. Treatment with H_2_O_2_ markedly increased the green fluorescence level in SH-SY5Y cells as an indicator of increased intracellular ROS levels. Co-treatment with 10 µM CI effectively attenuated this increase and reduced ROS production (Figure 4). 

### 3.5. CI Treatment Enhances the Expression of Anti-Apoptotic and Survival Proteins after H_2_O_2_ Stimulation in SH-SY5Y Cells

**Figure 5:**
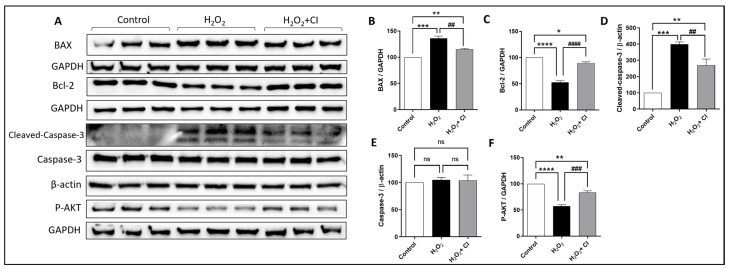
The effect of CI on BAX, Bcl-2, Caspases, and p-Akt expression in H_2_O_2_-stimulated SH-SY5Y cells. SH-SY5Y cells were treated with 200 μM H_2_O_2_ with or without 10 μM CI for 5 h; then, the cell culture medium was replaced with a fresh one with or without 10 μM CI for another 24 h. (**A**) WB immunoblots of BAX, Bcl-2, and p-Akt proteins in the Sh-SY5Y cell lysate. (**B**,**D**) Analysis of WB shows that the expression of BAX and cleaved caspase-3 were significantly upregulated in the H_2_O_2_ group compared to the control and were downregulated in the CI-treated group. (**E**) The expression of total caspase-3 showed no significant change in all groups. (**C**,**F**) Treatment with CI increases both Bcl-2 and p-Akt protein expression significantly compared to the H_2_O_2_-stimulated control. Data are expressed as mean ± SEM of three independent experiments where *p* < 0.05 was considered significant. * *p* < 0.05, ** *p* < 0.01, *** *p* < 0.001, **** *p* < 0.0001 relative to control group. ## *p* < 0.01, ### *p* < 0.001, #### *p* < 0.0001 relative to H_2_O_2_ group. (See Figure S4 for original Western blot images).

Cell survival, proliferation, and apoptosis are regulated by many factors, including the Bcl-2 family proteins, transcription factors, caspases, and Akt signaling pathway [29]. Thus, we used WB to investigate the protein expression changes of pro- and anti-apoptotic proteins in SH-SY5Y cells after H_2_O_2_ stimulation with or without CI treatments. As shown in Figure 5, CI treatment significantly attenuated the increased expression of BAX, a pro-apoptotic protein, and recovered the diminished expression of Bcl-2, an anti-apoptotic protein, in SH-SY5Y cells (Figure 5A–C). In addition, the expression of cleaved caspase-3, as a marker of cell apoptosis, was significantly suppressed in CI-treated cultures, while there was no difference in the protein expression of total caspase-3 (Figure 5A,D,E). Moreover, the reduced expression of p-Akt in the H_2_O_2_-stimulated cells was significantly rescued by CI treatment (Figure 5A,F). These observations demonstrate that cofilin activation mediated mitochondrial dysfunction and apoptotic cell death in models of oxidative stress in the SH-SY5Y cells.

### 3.6. CI Treatment Induces the Expression of Nrf2 and Antioxidant Enzymes in SY-SY5Y Cells

**Figure 6:**
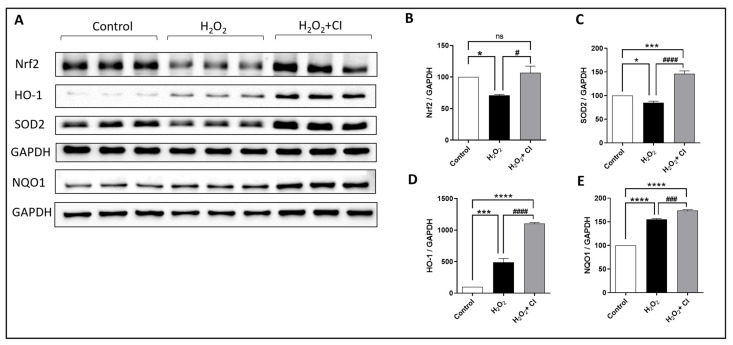
The effect of CI on the H_2_O_2_-induced oxidative stress in SH-SY5Y cells. SH-SY5Y cells were treated with 200 μM H_2_O_2_ with or without 10 μM CI for 5 h, then after the cell culture medium was replaced with a fresh one with or without 10 μM CI for another 24 h. (**A**) WB of different oxidative stress and antioxidant-related proteins in the Sh-SY5Y cell lysate was assessed. H_2_O_2_ significantly reduces the expression of Nrf2 (**B**) and SOD2 (**C**) and increases the expression of HO-1 (**D**) and NQO1 (**E**) compared to the unstimulated control group. However, in CI treatment groups, the levels of Nrf2, SOD2, HO-1, and NQO1 were significantly increased as compared to the H_2_O_2_ group. Data are expressed as mean ± SEM of three independent experiments where *p* < 0.05 was considered significant. * *p* < 0.05, *** *p* < 0.001, **** *p* < 0.0001 relative to control group. # *p* < 0.05, ### *p* < 0.001, #### *p* < 0.0001 relative to H_2_O_2_ group. (See Figure S5 for original Western blot images).

Nrf2 is a major transcription factor that protects against oxidative stress damage due to various diseases by regulating the expression of several cytoprotective enzymes [30]. Therefore, we tested the effect of CI on the expression of Nrf2 and relevant antioxidant enzymes, including SOD2, HO-1, and NQO1. In brief, SH-SY5Y cells were incubated with 200 μm H_2_O_2_ with or without 10 μM CI for 24 h; then, protein expression levels were measured (Figure 6A). Results from this experiment revealed that the reduced expression of Nrf2 and SOD2 by H_2_O_2_ was significantly recovered by CI treatment (Figure 6B,C). Moreover, the expression levels of HO-1 and NQO1, major Nrf2-dependent enzymes, were increased in the H_2_O_2_-treated cultures and were further increased when treated with CI (Figure 6D,E). Hence, these findings imply that activation of Nrf2 and its downstream antioxidant enzymes is a possible mechanism through which cofilin inhibition blocks oxidative damage in neuronal cells.

### 3.7. CI treatment Attenuates TBI-Induced Oxidative and Nitrosative Stress in Mice 

**Figure 7:**
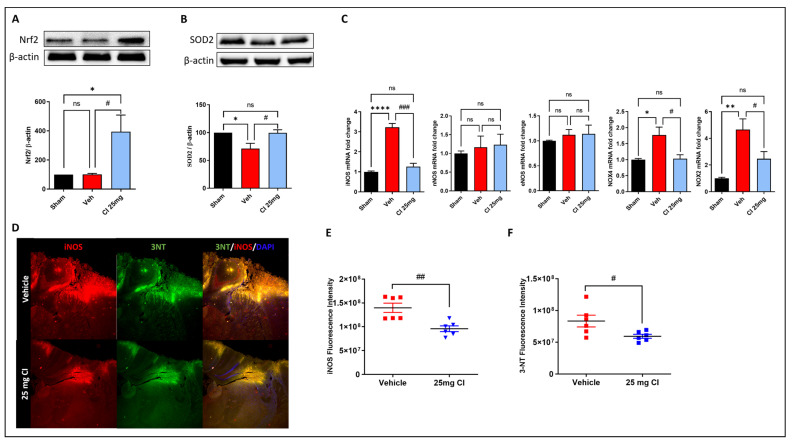
The effect of CI treatment on oxidative stress damage after TBI. CI increased the expression of Nrf2 (**A**) and SOD2 (**B**) in the perilesional cortex after TBI. (**C**) mRNA expression of oxidative stress markers determined by RT-qPCR showed a significant reduction in the gene expression of iNOS, NOX2, and NOX4, while there was no change in the levels of nNOS and eNOS. (**D**) Representative immunofluorescence images and quantitative analysis showed that CI significantly reduced the fluorescence intensity of iNOS (**E**) and 3-NT (**F**) in the perilesional cortex after TBI. (*n* = 6 per group) Data are expressed as mean ± SEM where *p* < 0.05 was considered significant. * *p* < 0.05, ** *p* < 0.01, **** *p* < 0.0001 relative to control group. # *p* < 0.05, ## *p* < 0.01, ### *p* < 0.001 relative to H_2_O_2_ group. (See Figure S6 for original Western blot images).

Oxidative stress is one of the major mechanisms that contributes to post-traumatic brain injury [8]. Thus, to further validate the efficacy of CI in rescuing oxidative stress in vivo, we tested the gene and protein expression of many oxidative stress regulators in mouse brain lysate 3 days following controlled cortical impact brain injury with or without 25 mg/kg of CI treatment. The results showed that the expression of Nrf2 was dramatically increased in the CI-treated mice compared to the vehicle and sham mice (Figure 7A). The TBI-induced reduction in SOD2 expression was rescued in the CI-treated mice (Figure 7B). Furthermore, we analyzed the gene expression of NADPH oxidases (NOX), nitric oxide synthases (NOS), and 3-nitrotyrosine (3-NT). qPCR analysis shows a significant elevation in the mRNA levels of inducible NOS (iNOS), NOX2, and NOX4, while no change was found in neuronal NOS (nNOS) and endothelial NOS (eNOS) (Figure 7C).

CI treatment significantly reduced the mRNA levels of iNOS, NOX2, NOX4 compared to the vehicle-treated mice. Additionally, immunofluorescence staining of iNOS, and 3-NT confirmed those results on the protein level and showed a significant reduction in the mean fluorescence intensity of iNOS and 3-NT in the traumatized cortex area of the CI-treated group in comparison with the vehicle group (Figure 7D–F). These observations confirm a relevant involvement of cofilin in brain injury-induced oxidative damage in mice.

## 4. Discussion

In the present study, we evaluated the role of cofilin in mediating oxidative stress-induced microglial activation and neurotoxicity and the potential therapeutic benefits of a novel small molecule cofilin inhibitor in vitro and in vivo. We demonstrated that microglial stimulation with H_2_O_2_ activates the cofilin pathway and increases the expression of inflammatory mediators such as TNFα and HMGB1, an effect reversed by treatment with CI. Moreover, we showed that CI increases the viability of SH-SY5Y cells and attenuates oxidative damage by altering the expression of apoptotic modulators and by activating the Nrf2 pathway, respectively. As a major TBI-induced secondary brain injury mechanism, oxidative damage was reduced in CI-treated mice on the gene and protein levels. Results from this study highlight the role of cofilin in mediating oxidative damage and propose the cofilin inhibitor as a promising therapeutic alternative for neurodegenerative diseases such as TBI.

The pathophysiology of traumatic brain injury is intrinsically heterogeneous and is mediated by multiple mechanisms, including ROS formation and oxidative damage, glutamate excitotoxicity, neuroinflammation, blood–brain barrier disruption, and cellular apoptosis [31]. Among these, neuroinflammation is considered one of the most critical mediators that orchestrates the pathology and chronic neurodegeneration processes during TBI [32]. Following TBI, microglia respond within minutes by becoming activated, migrating to the site of injury and mediating pathological responses, mainly the overexpression of inflammatory and oxidative/nitrosative stress factors, which aggravate the initial injury [32,33]. Thus, interventions targeting microglial activation are conceived to be potential neuroprotective therapeutic strategies for the treatment of TBI [34]. 

Cofilin is expressed in all cell types, and disturbance of cofilin dynamics has been involved in the pathology of many neurodegenerative disorders [35]. Cofilin is regulated by phosphatases such as SSH-1 that are activated in response to stress signals, such as ROS accumulation and ATP reduction, leading to cofilin dephosphorylation and activation [36,37]. In accordance with this, the present study shows that microglial stimulation with H_2_O_2_ activates cofilin through SSH-1 overexpression. Our previous studies on cofilin dynamics in microglia identified cofilin as a stress-associated protein that plays a pivotal role in microglial activation during pathological states. In this paradigm, cofilin knockdown by siRNA reduces LPS-induced microglial activation, proliferation, phagocytosis, and inflammatory cytokines production [16]. Moreover, treatment with a first-in-class cofilin inhibitor attenuates microglial activation and nitric oxide and TNFα expression in an in vitro model of hemorrhagic stroke [26]. In microglia, H_2_O_2_ insult promotes inflammatory phenotype represented by increased inflammatory protein levels such as TNFα, HMGB1, and IL-17 [17]. HMGB1 binds to microglial cellular receptors such as toll-like receptors (TLRs) that trigger the activation and translocation of NF-κB to the nucleus and the subsequent release of cytokines such as TNFα [38]. Similarly, the increase in HMGB1 and TNFα expression by H_2_O_2_ in this study confirms microglial activation, which was attenuated by CI treatment. However, there was no change in the level of total NF-κB. These findings are in agreement with other reports that suggest during a neuroinflammatory response, cofilin might intervene and promote the cytoskeleton dynamics of microglia and change its morphology into the activated ameboid phenotype [35,39].

We previously reported that inhibiting cofilin restores the viability of neuronal cells subjected to oxygen-glucose deprivation (OGD) or thrombin-induced cell death [25,26]. Furthermore, a study showed recently that cofilin knockout in primary cortical neurons enhances cell viability in a glutamate-induced excitotoxicity model [40]. In this study, although cofilin expression was not increased in SH-SY5Y cells stimulated with H_2_O_2_, the cofilin/p-cofilin ratio increased, indicating cofilin activation. To further explore whether inhibiting cofilin protects against oxidative-induced neuronal cell death, we investigated the effect of CI in H_2_O_2_-stimulated SH-SY5Y cells. We confirmed the neuroprotective effect of CI against H_2_O_2_-induced SH-SY5Y cell death by CCK-8 assay. 

It is evident that treating cells with H_2_O_2_ increases ROS production, which is associated with oxidative stress-induced DNA and mitochondrial damage, leading to cell apoptosis [41]. Here, we found that CI reduces ROS production in SH-SY5Y cells. These findings are consistent with a recent report proposing that inhibiting ROS accumulation by inhibiting cofilin is an effective intervention to salvage neurons under pathophysiological conditions such as glutamate or erastin stimulation [40]. Cofilin was identified as a key modulator in the apoptotic cell death cascade and was involved in transducing apoptotic signals to mitochondria during oxidative damage [42]. In this context, cofilin knockdown using siRNA inhibits H_2_O_2_-induced apoptosis in fibroblast cells [42]. Another study suggested that Aβ oligomers induce apoptosis by activating and translating cofilin to mitochondria regulated by SSH-1 [43]. A large body of evidence demonstrated that Akt promotes survival signals by regulating different protein kinases, transcription factors, and the expression of pro- and anti-apoptotic proteins, such as the Bcl-2 family [44]. Additionally, Akt activation has been suggested to be linked to apoptosis inhibition in SH-SY5Y cells under H_2_O_2_-induced oxidative damage [45]. As a member of the Bcl-2 family, BAX acts as a pro-apoptotic protein that induces the release of cytochrome c by impairing the mitochondrial outer membrane barrier function. On the opposite side, Bcl-2 is an anti-apoptotic protein that promotes mitochondrial membrane stabilization and inhibits the release of apoptogenic factors [46]. In the present study, H_2_O_2_ treatment upregulated BAX and cleaved caspase-3 expression, downregulated Bcl-2 expression, and reduced Akt phosphorylation in SH-SY5Y cells, of which the findings are consistent with previous studies [47,48]. However, these changes were significantly blunted in the presence of CI, proposing that CI may protect neuronal cell apoptosis by inhibiting mitochondrial damage-associated apoptotic regulators activated by oxidative stress.

As an adaptive cellular response to ROS accumulation and oxidative stress, Nrf2 is activated and induces the transcription of several antioxidant enzymes [13]. Several studies have shown that the activation of the Nrf2 signaling pathway is mainly responsible for neuroprotective effects from cytotoxic damage [47]. Additionally, we have previously shown that cofilin knockdown increases the expression of Nrf2 and HO-1 in hemin-treated microglial cells [24]. In line with these results, our data showed that CI induces the expression of Nrf2 and downstream antioxidant enzymes, including HO-1, SOD2, and NQO1 in H_2_O_2_-stimulated SH-SY5Y cells, which correlate with the ROS reduction shown earlier.

To further explore the potential therapeutic effect of CI against oxidative damage, we tested its efficacy in a TBI mouse model. The activation of Nrf2 is suggested to be crucial to offset oxidative damage after TBI through modulating mitochondrial and NAPDH oxidase functions [49]. Given this, an exacerbated neurological impairment accompanied by increased levels of oxidative stress and neuroinflammatory markers was reported in Nrf2 knockout mice following CCI injury [50]. On the other hand, TBI studies on NOX2 knockout mice showed reduced lesion volume and neuronal cell death, which were associated with downregulated gene expression of oxidative and pro-inflammatory markers such as iNOS, 3-NT, CD16, and CD86 [51,52]. Consistent with these lines of evidence and the in vitro data presented in this study, CI treatment enhanced the expression of Nrf2 and SOD2 and suppressed oxidative stress damage exhibited by the reduction of iNOS, NOX2, and NOX4 at the gene level, and iNOS and 3-NT at the protein level. These results align with an earlier study from our lab where localized knockdown of cofilin improved outcomes following intracerebral hemorrhage in mice partly through reducing oxidative and nitrosative stress [53]. These findings reveal that one of the potential mechanisms underlying the antioxidative effect of CI is through activating the Nrf2 signaling pathway.

## 5. Conclusions

**Figure 8:**
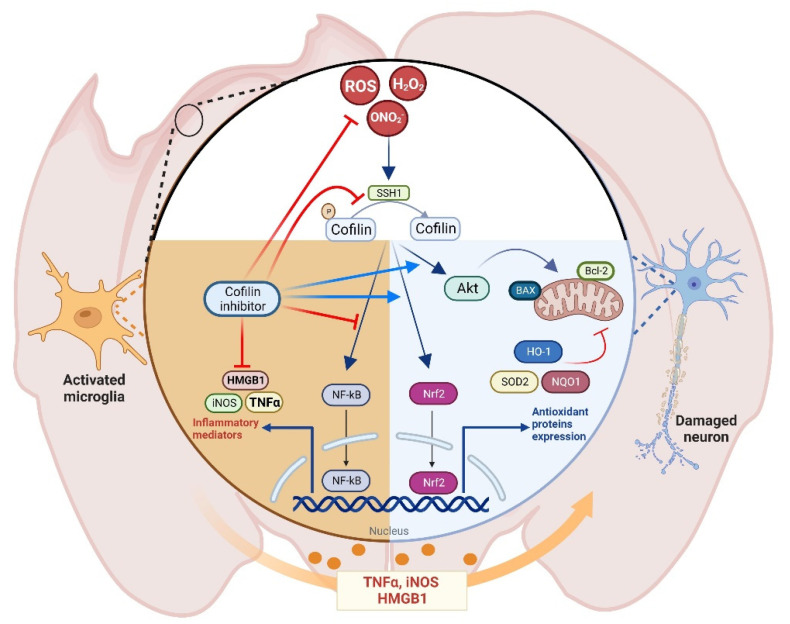
The proposed mechanism of cofilin inhibitor in TBI. Cofilin signaling is activated during oxidative stress conditions in microglial and neuronal cells. In microglia, ROS activates the NF-kB pathway, which regulates the expression of different cytotoxic mediators such as TNFα, HMGB1, and iNOS, an effect that is augmented by cofilin inhibitor treatment. In neurons, cofilin inhibitor activates the Nrf2 pathway, which regulates the expression of antioxidant enzymes and increases the Akt phosphorylation, leading to reduced expression of mitochondrial apoptotic markers and reduced neurotoxicity.

In conclusion, in the present study, the protective effect of CI against H_2_O_2_-induced microglial activation and neuronal cell death was elucidated, and some potential mechanisms were explored in vitro and in vivo (Figure 8). CI suppressed the oxidative-mediated microglial activation, represented by increased neuroinflammatory markers expression, via downregulating cofilin and SSH1. Additionally, CI retrieved neuronal viability and reversed ROS generation and mitochondrial dysfunction. In this process, CI modulated mitochondrial-related survival and apoptotic markers and activated Nrf2 and its related antioxidant enzymes, likely contributing to mitigating oxidative stress caused by H_2_O_2_ or TBI. Accordingly, the current findings confirm the role of cofilin and its targeting as a potential therapeutic strategy for the treatment and prevention of oxidative stress associated with different neurodegenerative diseases, particularly TBI. Future animal studies are required to investigate the role of cofilin in secondary TBI-induced brain damage and functional recovery and to evaluate the long-term effects of CI as a potential treatment of TBI.

## Figures and Tables

**Table 1:** Quantitative RT-PCR primers sequences.

Gene	Forward Primer	Reverse Primer
GAPDH	CTGGTGCGAAGTGTGCAAG	TGAGATTAGCGTGGCCCGAA
iNOS	CCAAATCCAACGTTCTCCGT	CCAAATCCAACGTTCTCCGT
eNOS	CAGATGCCCAACCCAAACCT	ACAGAGAGGTGTCTGGGACT
nNOS	CCTTCACAGGGGATGGAACC	AGATCGACAGCTTTGGTGGG
NOX2	CCCTCCCTGTCTAGGTAATGCATGG	GCATTTGCCTTCGGTGATGTGCT
NOX4	CACCAAATGTTGGGCGATTGT	GATGAGGCTGCAGTTGAGGT

## Data Availability

No data were used for the research described in the article.

## Supplementary Materials

The following supporting information can be downloaded at: https://www.mdpi.com/article/10.3390/biology12040630/s1, Figure S1: Original western blot for Figure 1 showing the bands with molecular weight markers. Figure S2: Original western blot for Figure 2 showing the bands with molecular weight markers. Figure S3: Original western blot for Figure 3 showing the bands with molecular weight markers. Figure S4: Original western blot for Figure 5 showing the bands with molecular weight markers. Figure S5: Original western blot for Figure 6 showing the bands with molecular weight markers. Figure S6: Original western blot for Figure 7 showing the bands with molecular weight markers.
