# Supplementary material for: Cofilin Inhibitor Protects against Traumatic Brain Injury-Induced Oxidative Stress and Neuroinflammation
Source: Biology (Basel). 2023 Apr 21;12(4):630. doi: 10.3390/biology12040630 (PMC10136258; doi:10.3390/biology12040630)
Supplement: Supplementary file 1 [file biology-12-00630-s001.zip › biology-2286972-Supplementary Material.pdf]

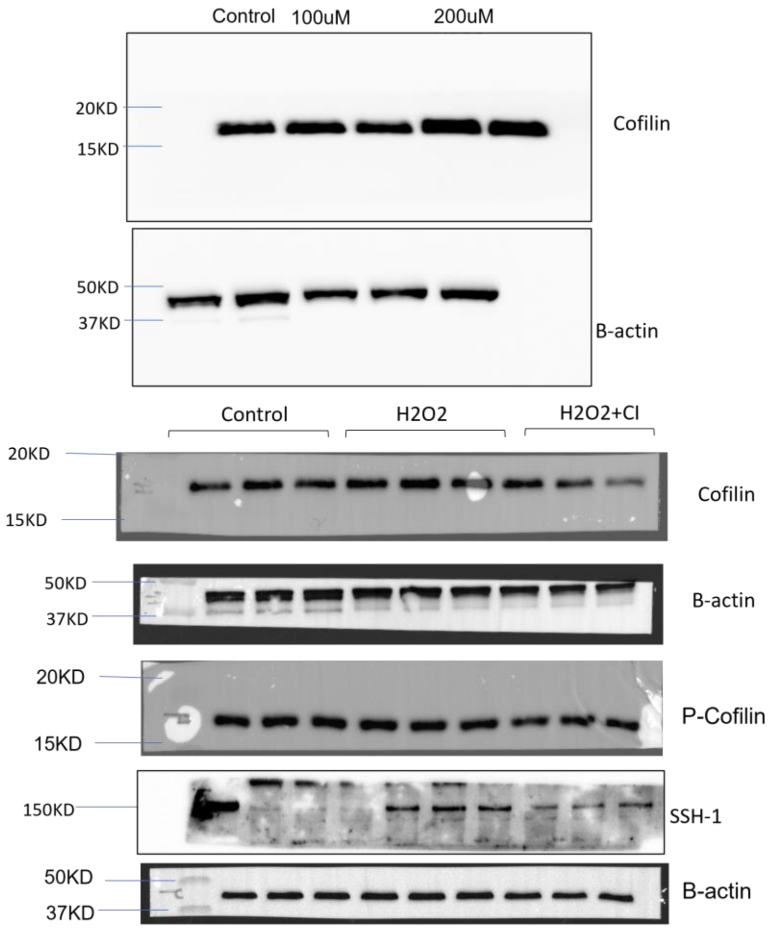

Figure S1: Original western blot for Figure 1 showing the bands with molecular weight markers.

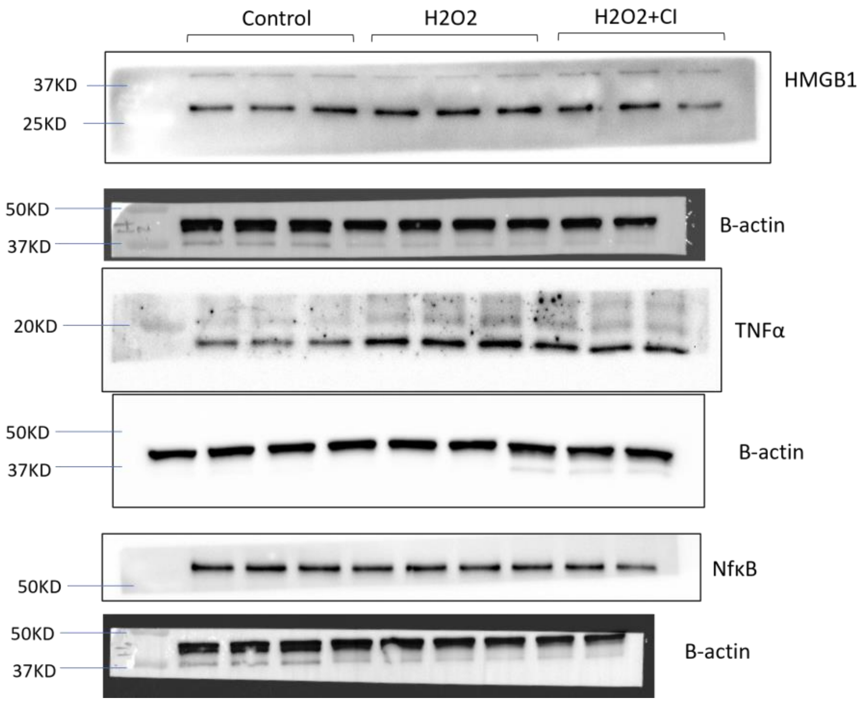

Figure S2: Original western blot for Figure 2 showing the bands with molecular weight markers.

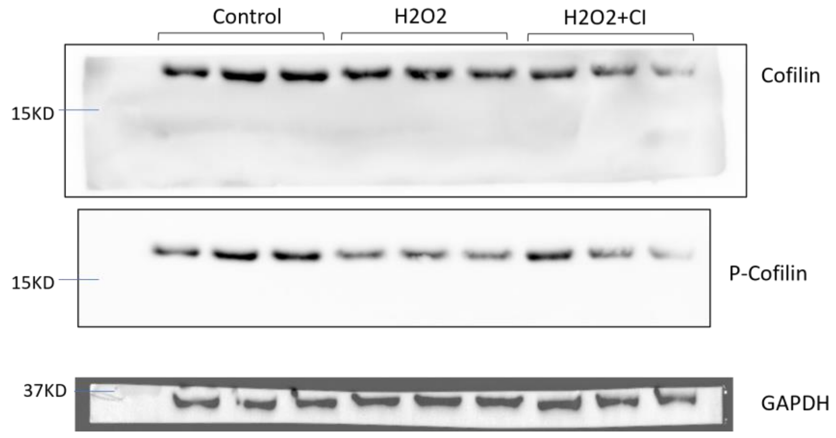

Figure S3: Original western blot for Figure 3 showing the bands with molecular weight markers.

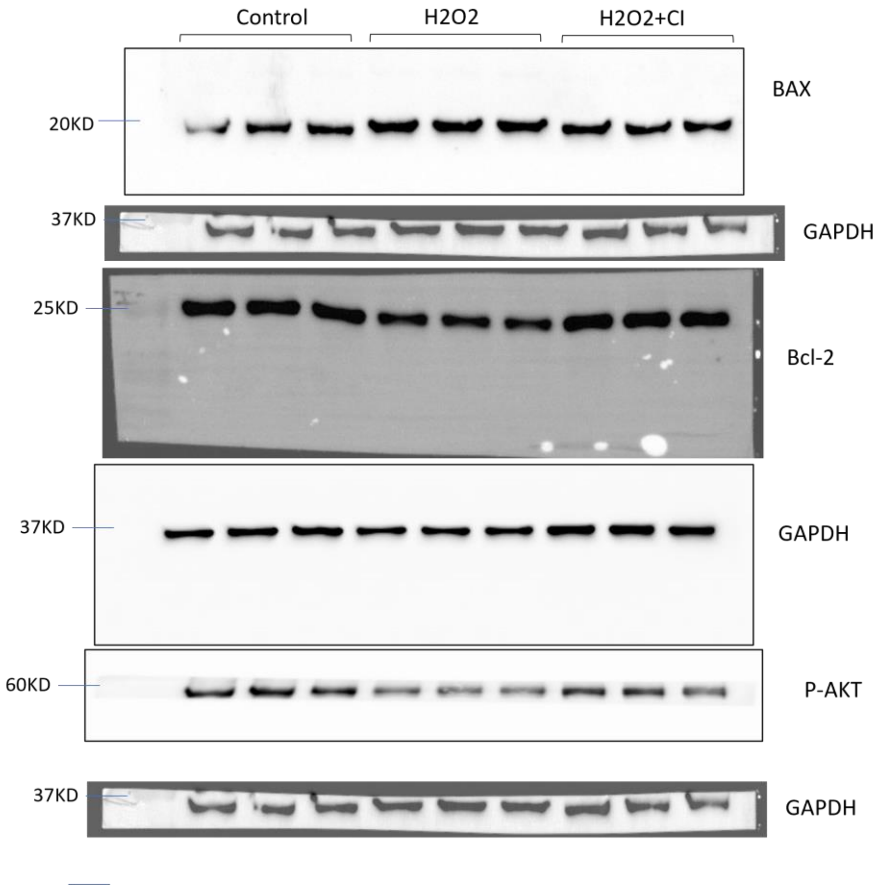

Figure S4: Original western blot for Figure 5 showing the bands with molecular weight markers.

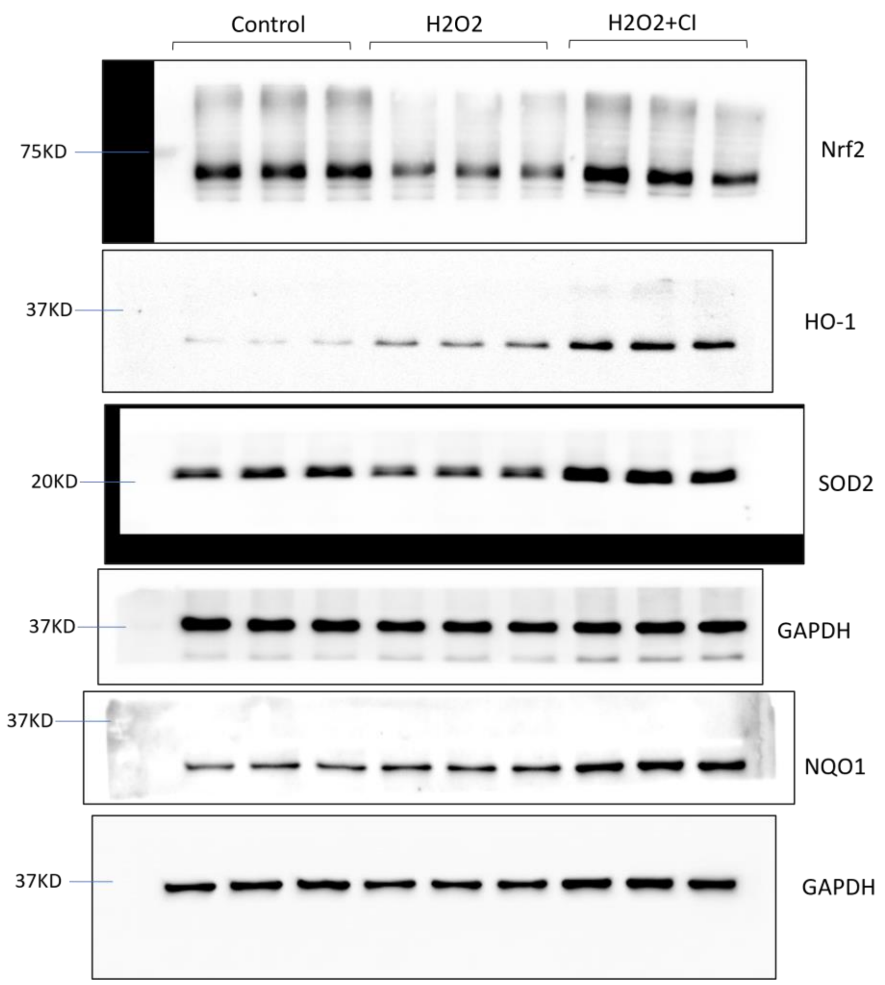

Figure S5: Original western blot for Figure 6 showing the bands with molecular weight markers.

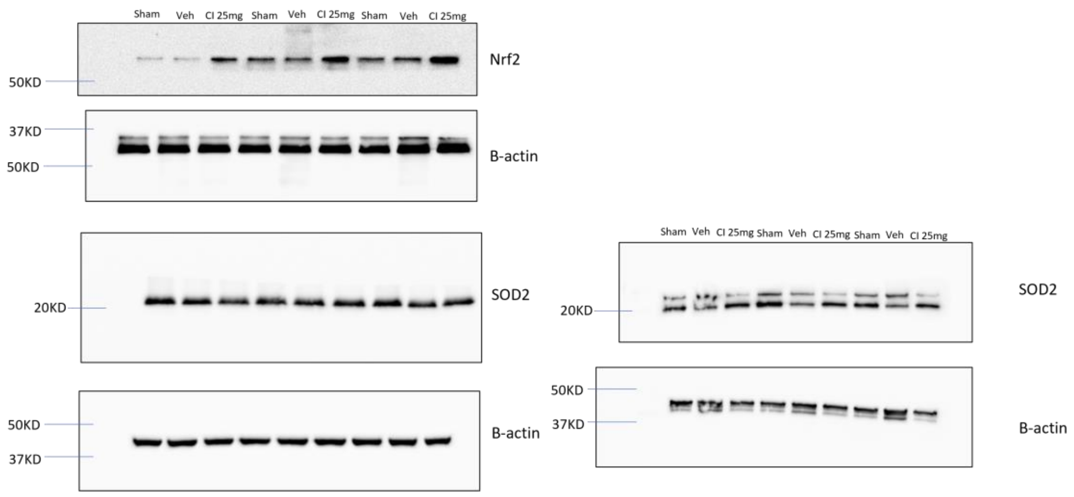

Figure S6: Original western blot for Figure 7 showing the bands with molecular weight markers.
